# Bone marrow transplantation reduces FGF-23 levels and restores bone formation in myelodysplastic neoplasms

**DOI:** 10.1038/s41375-024-02315-6

**Published:** 2024-06-21

**Authors:** Heike Weidner, Ulrike Baschant, Maria G. Ledesma-Colunga, Karolina Basiak, Elena Tsourdi, Katja Sockel, Katharina S. Götze, Jennifer Rivière, Uwe Platzbecker, Lorenz C. Hofbauer, Martina Rauner

**Affiliations:** 1 Department of Medicine III & Center for Healthy Aging, Medical Faculty and University Hospital Carl Gustav Carus, Dresden University of Technology, Dresden, Germany; 2 Department of Medicine I, Medical Faculty and University Hospital Carl Gustav Carus, Dresden University of Technology, Dresden, Germany; 3 Department of Medicine III, Technical University of Munich, School of Medicine and Health, Munich, Germany; 4 German Cancer Consortium (DKTK), partner sites Dresden and Munich and German Cancer Research Center (DKFZ), Heidelberg, Germany; 5 Bavarian Center for Cancer Research (BZKF), Munich, Germany; 6 Medical Clinic and Policlinic I, Hematology and Cellular Therapy, University Hospital Leipzig, Leipzig, Germany

**Keywords:** Cancer microenvironment, Haematopoietic stem cells, Myelodysplastic syndrome

## To the Editor:

Myelodysplastic neoplasms (MDS) are hematopoietic stem cell disorders characterized by ineffective hematopoiesis and dysplastic cells in the bone marrow (BM) [1]. In addition, patients with MDS display an increased susceptibility to osteoporosis [2]. Evidence points towards dysregulation in the BM niche that concurrently impairs bone turnover and hematopoiesis. We identified fibroblast growth factor (FGF)-23 as a critical regulator of bone mineralization and erythropoiesis. FGF-23 serum levels were higher in both patients and mice with MDS, and its neutralization resulted in improved erythropoiesis and bone mineralization in NUP98/HOXD13 (NHD13) mice [3]. FGF-23 is mainly produced by osteoblasts/osteocytes [4] and exerts phosphaturic effects leading to poor bone mineralization [5]. However, in NHD13 mice, intact FGF-23 (iFGF-23) and C-terminal FGF-23 (cFGF-23; produced by the cleavage of the intact form) protein levels were unchanged in the bone tissue, but erythroid progenitors secreted more FGF-23 compared to littermate wild-type (WT) controls (Fig. S1A, B).

**Fig. 1:**
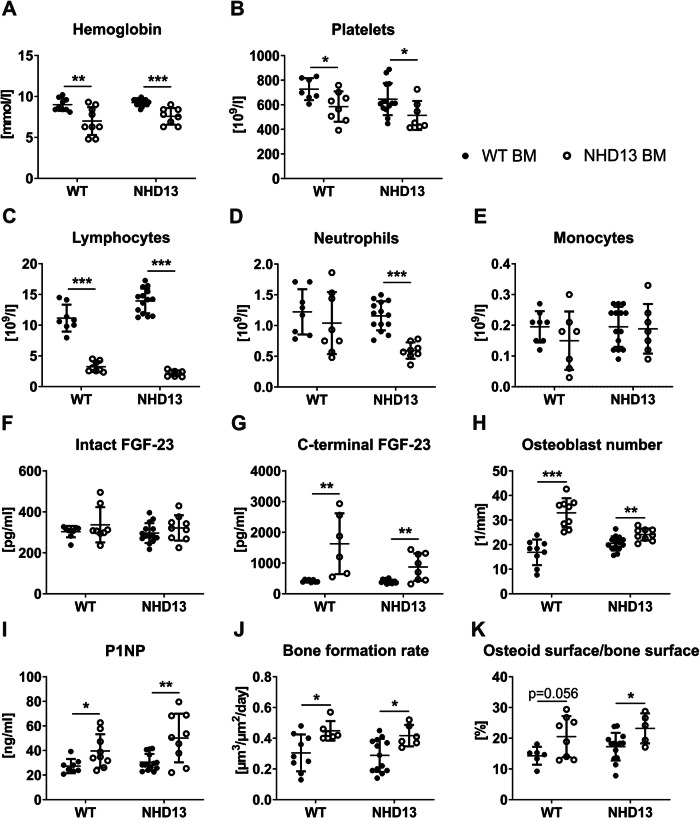
NHD13 bone marrow increases serum cFGF-23 and induces the MDS bone phenotype. Eigth-week-old male wild-type (WT) and NUP98/HOXD13 (NHD13) mice were lethally irradiated one day before 2 × 106 total bone marrow cells of age-matched WT (WT BM) or NHD13 (NHD13 BM) donor mice were transplanted by intravenous injection. After 16 weeks all mice were sacrificed and analyzed. The blood count, (**A**) hemoglobin levels (WT BM → WT: *n* = 9; NHD13 BM → WT: *n* = 9; WT BM → NHD13: *n* = 14; NHD13 BM → NHD13: *n* = 8), (**B**) platelet number (WT BM → WT: *n* = 8; NHD13 BM → WT: *n* = 8; WT BM → NHD13: *n* = 14; NHD13 BM → NHD13: *n* = 7) as well as the number of (**C**) neutrophils, (**D**) lymphocytes (WT BM → WT: *n* = 8; NHD13 BM → WT: *n* = 8; WT BM → NHD13: *n* = 14; NHD13 BM → NHD13: *n* = 8), and (**E**) monocytes (WT BM → WT: *n* = 8; NHD13 BM → WT: *n* = 7; WT BM → NHD13: *n* = 15; NHD13 BM → NHD13: *n* = 7) were received using the Sysmex XN-100 (Sysmex, Norderstedt, Germany). After collecting the serum, (**F**) the intact (WT BM → WT: *n* = 9; NHD13 BM → WT: *n* = 8; WT BM → NHD13: *n* = 14; NHD13 BM → NHD13: *n* = 9) as well as (**G**) C-terminal fibroblast growth factor (FGF)-23 (WT BM → WT: *n* = 8; NHD13 BM → WT: *n* = 6; WT BM → NHD13: *n* = 13; NHD13 BM → NHD13: *n* = 8) were measured by ELISA. (**H**) The osteoblasts per bone perimeter were evaluated in TRAP-stained vertebral bone slices (WT BM → WT: *n* = 9; NHD13 BM → WT: *n* = 9; WT BM → NHD13: *n* = 14; NHD13 BM → NHD13: *n* = 9) and (**I**) the osteoblast activity was assessed by procollagen type I N-propeptide (P1NP) using ELISA (WT BM → WT: *n* = 8; NHD13 BM → WT: *n* = 9; WT BM → NHD13: *n* = 14; NHD13 BM → NHD13: *n* = 9). **J** To determine the bone formation rate in vertebrae, mice received intraperitoneal calcein injection 5 and 2 days before sacrifice for the double labeling analysis (WT BM → WT: *n* = 9; NHD13 BM → WT: *n* = 6; WT BM → NHD13: *n* = 13; NHD13 BM → NHD13: *n* = 6). **K** Embedded vertebrae were stained with von Kossa/van Gieson to determine the osteoid surface per bone surface (WT BM → WT: *n* = 6; NHD13 BM → WT: *n* = 8; WT BM → NHD13: *n* = 14; NHD13 BM → NHD13: *n* = 5). Data are shown as mean ± SD of five independent experiments. Statistical analysis was performed by two-sided Student´s *t* test. *p < 0.05; **p < 0.01; ***p < 0.001.

Here, we tested the hypothesis that erythroid precursors contribute to increased FGF-23 production/cleavage in MDS as a cause for impaired erythropoiesis and bone mineralization. To that end, we used BM transplantation as a first approach to substitute myelodysplastic erythroblasts with healthy ones in NHD13 mice. Four months after the BM transplantation, all mice that received the NHD13 BM showed MDS-like symptoms. In WT recipients, a reduction in hemoglobin levels [−32%; p < 0.001], platelets [−20%; p < 0.05], and lymphocytes [−71%; p < 0.001], but not in neutrophils or monocytes was observed compared to WT controls (transplanted with WT BM), showing a similar MDS status as NHD13 controls. In turn, NHD13 mice transplanted with WT BM did not develop MDS during the observation period. Compared to NHD13 controls, blood count reached normal levels [hemoglobin: +22%; p < 0.001; platelets: +26%; p < 0.05; lymphocytes: +6.5-fold; p < 0.001; neutrophils: +2-fold; p < 0.001] (Fig. 1A–E). This confirms that the MDS blood phenotype is transferable via hematopoietic cells. In line with NHD13 mice only showing increased cFGF-23 levels, but normal serum levels of iFGF-23 [3], the transplantation of WT or NHD13 BM into either WT or NHD13 recipient mice did not alter iFGF-23 (Fig. 1F). In contrast, cFGF-23 was increased in all recipients of NHD13 BM [WT: +3.5-fold; p < 0.05; NHD13: +2.1-fold; p < 0.01] compared to the corresponding mice with WT BM (Fig. 1G). Because of the transferable FGF-23 status, we hypothesized that WT mice receiving NHD13 BM would exhibit a bone phenotype mimicking the NHD13 controls. That was the case regarding the increased bone formation parameters usually observed in NHD13 mice. Similar to NHD13 mice, WT mice receiving NHD13 BM showed an increased number of osteoblasts [+95%; p < 0.001] concomitant with elevated levels of the bone formation marker procollagen type I N-propeptide [+45%; p < 0.05] and an increased bone formation rate [+87%; p < 0.01] (Fig. 1H–J). Also, the osteoid surface per bone surface tended to be increased [+44%; p = 0.056] (Fig. 1K). Importantly, transplanting WT BM to NHD13 mice normalized their bone formation parameters (Fig. 1H–K), indicating that hematopoietic cell signals control bone formation in NHD13 mice.

**Fig. 2:**
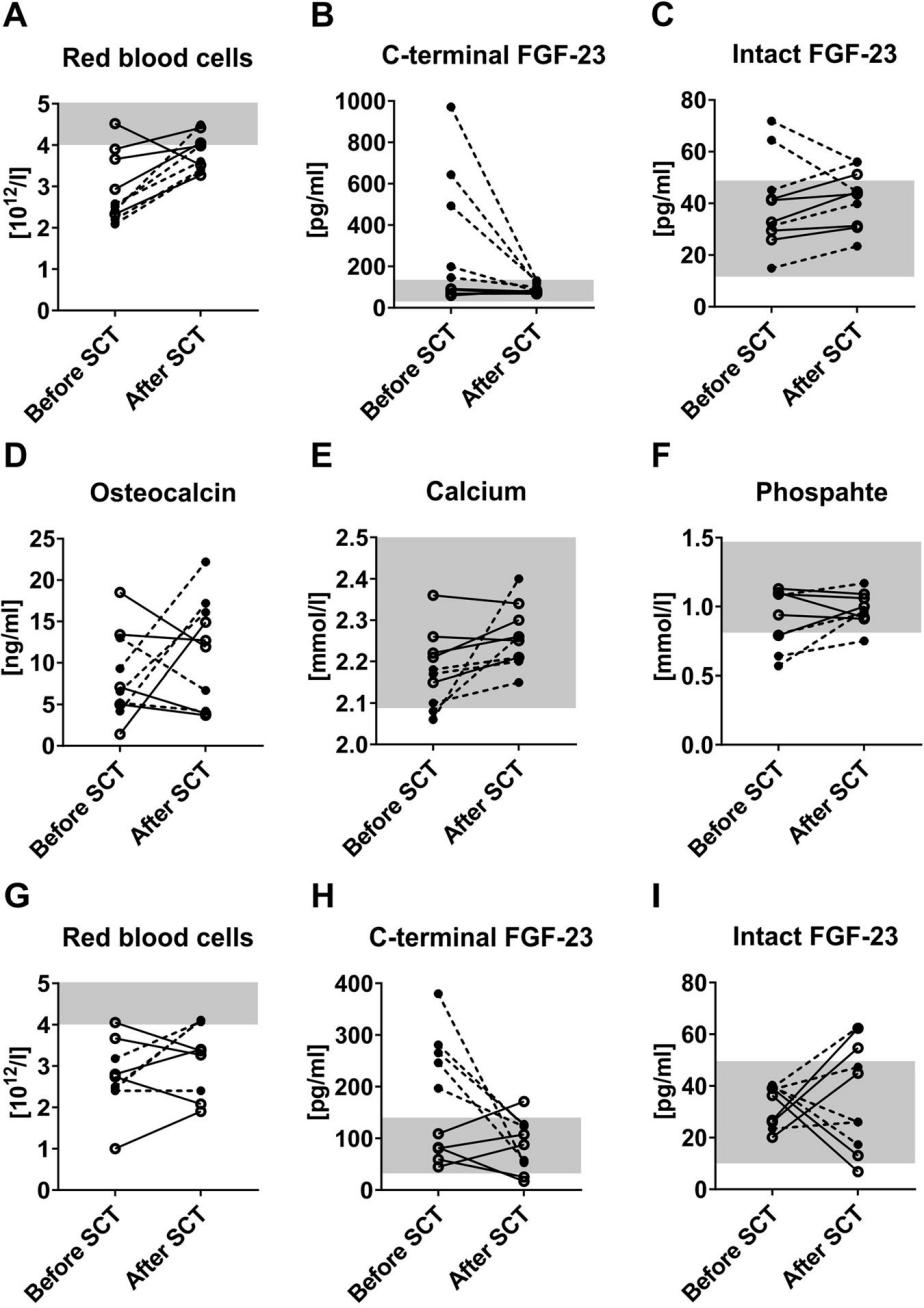
Normal cFGF-23 is linked with improved bone formation in patients with MDS after stem cell transplantation. The hematological and plasma parameters of patients with MDS were analyzed before and after allogeneic stem cell transplantation (SCT) in blood (**A**–**F**) and bone marrow samples (**G**–**I**). **A**, **G** The number of red blood cells was determined by the Sysmex XN-100 (Sysmex, Norderstedt, Germany). **B**, **H** C-terminal fibroblast growth factor (FGF)-23, (**C**, **I**) intact FGF-23, as well as (**D**) osteocalcin, were determined in plasma samples and in serum (**E**) albumin-adjusted calcium levels, as well as (**F**) phosphate levels, were measured by ELISA. *n* = 10 except (**G**) *n* = 9. The grey boxes in the graphs mark the reference range of healthy individuals. In all graphs, each dot represents a patient with MDS, and the values from the same patient are connected by a line (normal C-terminal FGF-23 before SCT, *n* = 5) or dotted line (high C-terminal FGF-23 before SCT, *n* = 5).

To address whether stem cell transplantation (SCT) leads to similar changes in FGF-23 in patients with MDS, we employed samples from the BoHemE study, in which we previously confirmed the high plasma iFGF-23 and cFGF-23 levels in patients with MDS [3]. Within this cohort, we identified 10 patients with MDS (3 women, 7 men; median age: 64 years; without renal disease) who had undergone SCT. We analyzed their hematological and bone-specific parameters before (range: 1–6 months) and after SCT (range: 5–11 months). SCT led to a higher number of red blood cells in 9/10 patients, neutrophils in 9/10 patients, and lymphocytes in 6/10 patients with normal monocyte counts. Platelet counts were below the reference range in 8/10 patients and higher in 1/10 patients before SCT, but only 4 patients had persistent thrombocytopenia after SCT (Figs. 2A,  S2A–D). Before SCT, 5 patients showed elevated cFGF-23 plasma levels, which were normalized after SCT (Fig. 2B). Elevated iFGF-23 levels were observed in 2 patients before SCT, with levels decreasing post-SCT. However, after SCT, iFGF-23 levels slightly increased in all patients with normal baseline levels (Fig. 2C). Additionally, 7/10 patients had reduced osteocalcin levels (bone formation) before SCT, though this did not result in abnormal bone mineral density (BMD) (Fig. 2D, Table S1). Given that bone mineralization is impaired in MDS [3], we also analyzed albumin-adjusted calcium, phosphate, and bone-specific alkaline phosphatase (BSAP). Calcium levels were reduced in 2/5 patients with elevated cFGF-23 and normalized after SCT (Fig. 2E). All patients with normal cFGF-23 had calcium levels within the reference range, with only one showing reduced levels of phosphate, which were corrected by the SCT. Whereas 3/5 patients with high cFGF-23 had mild to moderate hypophosphatemia before SCT, only one remained hypophosphatemic after SCT (Fig. 2F). In line with the increase of serum phosphate, BSAP levels, the major regulator of bone mineralization, also increased after SCT with high cFGF-23 (determined in 3/5 patients only, Fig. S2E). In addition, we analyzed 10 BM plasma samples regarding cFGF-23 and iFGF-23 levels in a separate set of patients with MDS (4 women, 6 men; median age: 57 years; Table S2). Since the samples were collected relatively shortly after SCT (range: 2–8 months), it is not surprising that the number of red blood cells was equal or decreased after SCT in 4/9 patients (data from one patient are not evaluable) compared to the basal levels (Fig. 2G). In line with our previous observations, before SCT 5/10 patients had elevated cFGF-23 levels, which normalized after SCT. In patients with normal baseline cFGF-23, levels were either slightly increased (1/5 patients) or decreased (2/5) after SCT (Fig. 2H). Before SCT, all patients had normal iFGF-23 levels. The SCT led to an increase in 6/10 patients and a decrease in 4/10 patients independently of the basal iFGF-23 levels (Fig. 2I). Overall, the regulation of cFGF-23 in blood and BM plasma samples from patients with MDS after SCT suggests that BM cells are a source for cFGF-23 in MDS. This is supported by the transplantation of NHD13 BM cells, which causes the increase of cFGF-23 levels leading to impaired erythropoiesis and bone mineralization. Only erythroid precursors of NHD13 mice show a high *Fgf23* expression, but myeloid cells and megakaryocytes do not (Fig. S1C, D). The question remains why and how cFGF-23 levels are increased in MDS. The expression of *Galnt3* and *Fam20c*, which stabilize or mark FGF-23 for cleavage [6, 7], was normal in NHD13 erythroid precursors (Fig. S1E, F), indicating that these cells do not directly contribute to the increased cleavage of erythroid-derived FGF-23. Therefore, other signals or cells within the bone microenvironment or beyond may participate in this regulation. In line with this, it has been shown that FGF-23 production/cleavage can be triggered by erythropoietin, iron deficiency, anemia, and inflammation [8–10], factors that also play a role in MDS [11, 12]. While iron serum levels are unchanged in NHD13 mice, erythropoietin is upregulated. Since erythropoietin can affect FGF-23 production/cleavage in WT erythroid cells [8], this may be a possible regulator also in NHD13 mice. The whole inflammatory status of NHD13 mice has not been described yet, suggesting that inflammation and/or anemia might be drivers of cFGF-23 in NHD13 mice as well. The production of cFGF-23 is increased by inflammation, inhibits hepcidin induction in the liver, and increases iron bioavailability independent of the functions of iFGF-23 [10]. This scenario may hold true for MDS as it is also characterized by inflammation (and anemia) and may require high levels of cFGF-23 to provide enough iron for erythropoiesis. In our human cohorts, not all patients with MDS showed elevated cFGF-23 levels, and not all patients with elevated cFGF-23 showed dysregulations of iron or inflammation (Table S1). All patients with elevated cFGF-23 however did have anemia. MDS is a heterogeneous group of disorders. As NHD13 mice mimic a severe form of MDS with a high percentage of blasts in the bone marrow and a high propensity to transformation towards acute leukemia [13], we included patients with intermediate to very high-risk MDS in our cohorts and indicated their mutations. Analyzing the mutational landscape in patients with MDS might further allow assumptions on the underlying mechanisms leading to increased cFGF-23 levels. Mutations like *TET2*, *DNMT3A*, *ASXL1, RUNX1*, *SF3B1*, and SRSF2 are linked to increased responses to inflammatory stimuli [12, 14], and *Tet2* or *Dnmt3a* deficiency causes bone loss in mice due to increased osteoclastogenesis [15]. In our blood plasma cohort, 4/5 patients with elevated cFGF-23 carried a mutation in at least one of these genes. However, 2/5 patients with normal cFGF-23 also had these mutations, but they received the MDS diagnosis a month earlier only. It is conceivable that the cFGF-23 levels increase after some time. Future research is needed to determine whether any of these mutations contribute to high cFGF-23 levels. In summary, we show that the high serum cFGF-23 levels in MDS originate from the BM and that BM transplantation/SCT can reduce cFGF-23 levels and its associated negative effects on erythropoiesis and bone mineralization. Future studies need to validate these findings in humans and address why cFGF-23 levels are increased in MDS.

### Supplementary information

Supplementary information

## Data Availability

The described methods and generated data of the current study are available from the corresponding author upon request.
