# Supplementary material for: Bone marrow transplantation reduces FGF-23 levels and restores bone formation in myelodysplastic neoplasms
Source: Leukemia. 2024 Jun 21;38(8):1853–7. doi: 10.1038/s41375-024-02315-6 (PMC11286507; doi:10.1038/s41375-024-02315-6)
Supplement: Supplementary file 1 — Supplementary information [file 41375_2024_2315_MOESM1_ESM.pdf]

## Supplementary information: Methods

### Murine bone marrow transplantation

All mouse experiments were approved by the institutional animal care committee and the Federal State of Saxony (TVV 64/2022). Mice were fed with a standard diet and water *ad libitum*, and exposed to a 12 h light/dark cycle in an air-conditioned room at 23°C as well as housed under institutional guidelines in the animal facility of Technische Universität Dresden.

Two-month-old wild-type (WT) and NUP98/HOXD13 (NHD13) mice were lethally irradiated with 7 Gy and transplanted with 2 x $10^6$ BM cells of age-matched WT or NHD13 donor mice by intravenous injection. The blood count was monitored monthly over four months. For the subsequent analysis of the bone formation rate, five and two days before sacrifice all donor mice were treated intraperitoneally with calcein (20 mg/kg body weight). At the end of the experiment, the vertebral bodies L1 and L2 were embedded in paraffin and 2 µm thick sections were used for tartrate-resistant acid phosphatase staining to assess the number of osteoblasts. The L3 and L4 were embedded in methyl methacrylate and 4 µm thick sections were stained with von Kossa/van Gieson to evaluate the osteoid surface per bone surface.

### Patient Cohort

Blood and bone marrow samples were collected at TU Dresden within the BoHemE study (Bone and Hematopoiesis in Elderly, NCT02867085) or at TU Munich. Both studies were approved by an internal review board. Written informed consent was obtained from all patients and data was treated according to the principles of the Declaration of Helsinki.

### Serum and plasma analysis

On the day of sacrifice, serum was collected from all mice to measure the intact as well as C-terminal FGF-23 (Quidel Corporation, San Diego, CA, USA) and procollagen type I N-propeptide (P1NP; IDS, Frankfurt/Main, Germany).

For humans, the plasma levels of intact as well as C-terminal FGF-23 (Quidel Corporation, San Diego, CA, USA) were measured in blood and bone marrow. Osteocalcin (IDS, Frankfurt/Main, Germany) was measured in blood plasma. Serum blood samples were used

for calcium, phosphate, and bone-specific alkaline phosphatase (all, IDS, Frankfurt/Main, Germany). All used ELISA are commercially available.

**Supplementary information:Tables**

**Table S1. Characteristics of patients with MDS (blood plasma) before autologous stem cell transplantation.**

| | Normal cFGF-23 | | | | | High cFGF-23 | | | | |
|---|---|---|---|---|---|---|---|---|---|---|
| | **Patient 1** | **Patient 2** | **Patient 3** | **Patient 4** | **Patient 5** | **Patient 6** | **Patient 7** | **Patient 8** | **Patient 9** | **Patient 10** |
| **MDS subtype** | EB2 | MLD | EB2 | EB2 | EB2 | EB1 | MLD | RS-MLD | EB1 | EB2 |
| **Sex** | m | m | m | m | f | m | m | m | f | f |
| **Diagnosis before screening [months]** | 1 | 1 | 1 | 40 | 16 | 17 | 8 | 3 | 11 | 17 |
| **Molecular genetics (mutations)** | ASXL1<br>CUX1<br>CEBPA<br>TET2<br>SF3B1<br>STAG | - | ASXL1<br>IDH2<br>KMT2A-PTD<br>SRSF2 | SMCA1 | BCOR<br>NKRAS | ASXL1<br>SF3B1<br>TET2 | EZH2<br>TET2<br>TP53 | ATRX<br>DNMT3A<br>CUX1<br>SF3B1<br>TET2 | ASXL1<br>RUNX1<br>FLT3-ITD<br>TP53 | BRAF |
| **Transfusion-dependent** | yes | no | no | no | no | yes | yes | no | no | yes |
| **Serum iron [µmol/l]*** | 36.9 | 39.8 | 20.6 | 19.9 | 24.4 | 15.7 | 46.4 | 39.2 | 18.2 | 19.8 |
| **Transferrin saturation [%]†** | 83.9 | 66.0 | 34.9 | 35.7 | 43.0 | 36.1 | 89.6 | 90.2 | 31.4 | 38.4 |
| **Serum ferritin [µg/l]‡** | 1483 | 878 | 584 | 342 | 280 | 311 | 2893 | 3019 | 175 | 1350 |
| **Osteoporosis** | no | no | no | no | no | no | § | no | yes | no |
| **Total hip BMD [g/cm$^2$]‖** | 1.235 | 0.921 | 1.030 | 1.042 | 0.986 | 0.796 | § | 0.900 | 0.696 | 0.868 |
| **Comorbidities** | - | - | RA<br>T2DM | Cancer<br>HT | T2DM | - | Cancer<br>T2DM<br>HT | Cancer<br>MM | HT | Cancer<br>HT |

Reference value: * 7.2 - 21.5 µmol/l; † 16 – 45%; ‡ 30 – 400 µg/l; ‖ World Health Organization, 2007

§ not evaluable; m: male; f: female; cFGF-23: C-terminal fibroblast growth factor-23; BMD: bone mineral density; RA: rheumatoid arthritis; T2DM: type 2 diabetes mellitus; HT: hypothyroidism; MM: multiple myeloma.

**Table S2. Characteristics of patients with MDS (bone marrow plasma) before autologous stem cell transplantation.**

| | Normal cFGF-23 | | | | | High cFGF-23 | | | | |
|---|---|---|---|---|---|---|---|---|---|---|
| | **Patient 1** | **Patient 2** | **Patient 3** | **Patient 4** | **Patient 5** | **Patient 6** | **Patient 7** | **Patient 8** | **Patient 9** | **Patient 10** |
| **MDS subtype** | EB2 | EB1 | EB2 | MLD | EB2 | EB1 | EB1 | EB1 | EB2 | EB1 |
| **Sex** | m | m | m | m | m | m | f | f | f | f |
| **Molecular genetics (mutations)** | ASXL1 | - | ASXL1<br>DNMT3A<br>RUNX1 | RUNX1<br>EZH2<br>PHF6 | SF3B1 | SRSF2<br>STAG2 | ASXL1<br>ETV6<br>RUNX1<br>SF3B1 | SETBP1<br>SRSF2 | - | ASXL1 |
| **Transfusion-dependent** | no | no | no | no | no | no | no | no | no | yes |
| **Comorbidities** | - | - | IDA | Cancer | T2DM<br>MGUS | T2DM | HMT | - | - | - |

§ not evaluable; m: male; f: female; cFGF-23: C-terminal fibroblast growth factor-23;IDA: iron deficiency anemia; T2DM: type 2 diabetes mellitus; MGUS: monoclonal gammopathy of undetermined significance; HMT: Hashimot thyreoditis.

**Supplementary information: Figure**

**Figure S1: FGF-23 is highly expressed in erythroid cells but normal in bone, myeloid cells, and megakaryocytes in MDS mice.** Six-month-old wild-type (WT) and NUP98/HOXD13 (NHD13) mice were used for protein extraction from flushed femora (bone) and erythroid cells (Ter119+ cells) or for RNA isolation from myeloid cells, megakaryocytes, and erythroid precursors. (A) Intact fibroblast growth factor (FGF)-23 (Bone: WT: *n*=9, NHD13: *n*=8; Ter119+ cells: WT: *n*=5, NHD13: *n*=5) and (B) C-terminal FGF-23 (Bone: WT: *n*=8, NHD13: *n*=8; Ter119+ cells: WT: *n*=5, NHD13: *n*=5) were measured by ELISA. The expression of *Fgf23* was assessed in (C) myeloid cells (WT and NHD13: *n*=5) and (D) megakaryocytes (WT: *n*=3, NHD13: *n*=4). The expression levels of (E) *Galnt3* (WT and NHD13: *n*=10) and (F) *Fam20c* (WT: *n*=11, NHD13: *n*=8) were determined in erythroid precursors. Data are shown as mean ± SD. Statistical analysis was performed by two-sided Student´s *t*-test. *$p < 0.05$.

**Figure S2: Normalization of C-terminal FGF-23 increases white blood cells and improves bone-specific alkaline phosphatase in patients with MDS.** Blood samples and serum were analyzed from patients with MDS before and after allogeneic stem cell transplantation (SCT). The number of (A) neutrophils, (B) lymphocytes, (C) monocytes, and (D) platelets was determined by Sysmex XN-100 (Sysmex, Norderstedt, Germany). n=10. The grey boxes in the graphs mark the reference range of healthy individuals. (E) The bone-specific alkaline phosphatase (BSAP) was measured by ELISA. n=8. In all graphs, each dot represents a patient with MDS, and the values from the same patient are connected by a line (normal C-terminal FGF-23 before SCT, n=5) or dotted line (high C-terminal FGF-23 before SCT, n=5).

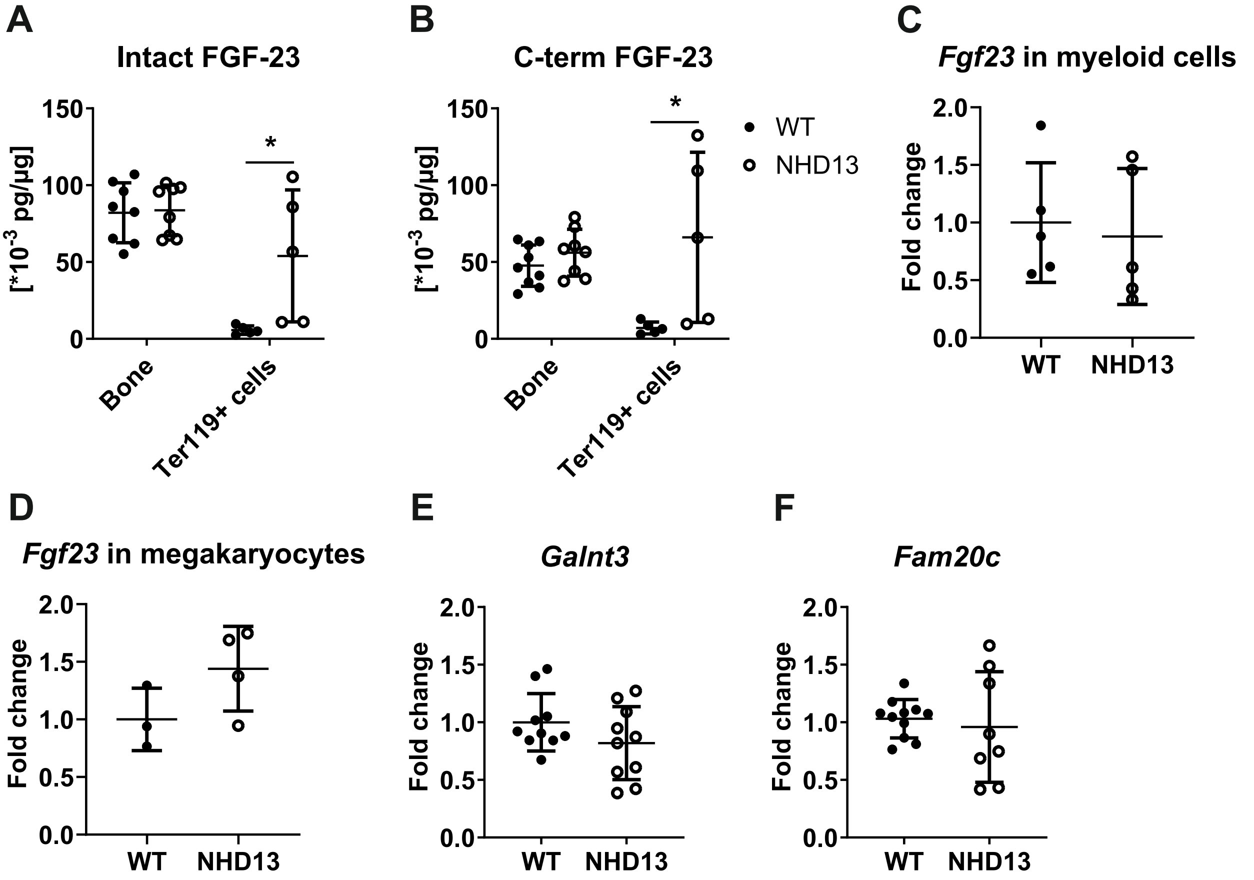
A
Intact FGF-23
[*10-3 pg/µg]
150
100
50
0
*
Bone
Ter119+ cells
B
C-term FGF-23
[*10-3 pg/µg]
150
100
50
0
*
Bone
Ter119+ cells
WT
NHD13
C
Fgf23 in myeloid cells
Fold change
2.0
1.5
1.0
0.5
0.0
WT
NHD13
D
Fgf23 in megakaryocytes
Fold change
2.0
1.5
1.0
0.5
0.0
WT
NHD13
E
Galnt3
Fold change
2.0
1.5
1.0
0.5
0.0
WT
NHD13
F
Fam20c
Fold change
2.0
1.5
1.0
0.5
0.0
WT
NHD13

**Figure S1**

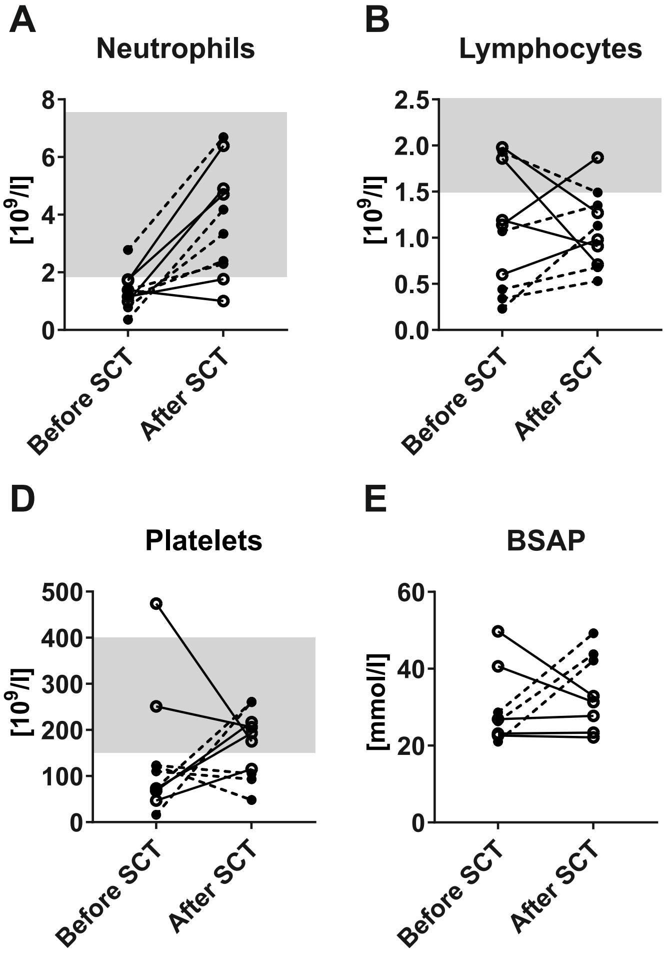
A
Neutrophils
[10^9/l]
0
2
4
6
8
Before SCT
After SCT
B
Lymphocytes
[10^9/l]
0.0
0.5
1.0
1.5
2.0
2.5
Before SCT
After SCT
C
Monocytes
[10^9/l]
0.0
0.2
0.4
0.6
0.8
1.0
Before SCT
After SCT
D
Platelets
[10^9/l]
0
100
200
300
400
500
Before SCT
After SCT
E
BSAP
[mmol/l]
0
20
40
60
Before SCT
After SCT

**Figure S2**
